# Provider volume and maternal complications after Caesarean section: results from a population-based study

**DOI:** 10.1186/s12884-019-2709-5

**Published:** 2020-01-14

**Authors:** Philip S. J. Leonard, Dan L. Crouse, Jonathan G. Boudreau, Neeru Gupta, James T. McDonald

**Affiliations:** 1 Department of Economics, University of New Brunswick, Singer Hall, Room 459, P.O. Box 4400, Fredericton, New Brunswick E3B 5A3 Canada; 2 New Brunswick Institute for Research, Data, and Training, University of New Brunswick, Fredericton, New Brunswick Canada; 3 Department of Sociology, University of New Brunswick, Fredericton, New Brunswick Canada

**Keywords:** Caesarean section, Postoperative complications, Epidemiological research design, Maternal health

## Abstract

**Background:**

A large literature search suggests a relationship between hospital/surgeon caseload volume and surgical complications. In this study, we describe associations between post-operative maternal complications following Caesarean section and provider caseload volume, provider years since graduation, and provider specialization, while adjusting for hospital volumes and patient characteristics.

**Methods:**

Our analysis is based on population-based discharge abstract data for the period of April 2004 to March 2014, linked to patient and physician universal coverage registry data. We consider all hospital admissions (*N* = 20,914) in New Brunswick, Canada, where a Caesarean Section surgery was recorded, as identified by a Canadian Classification of Health Intervention code of 5.MD.60.XX. We ran logistic regression models to identify the odds of occurrence of post-surgical complications during the hospital stay.

**Results:**

Roughly 2.6% of admissions had at least one of the following groups of complications: disseminated intravascular coagulation, postpartum sepsis, postpartum hemorrhage, and postpartum infection. The likelihood of complication was negatively associated with provider volume and provider years of experience, and positively associated with having a specialization other than maternal-fetal medicine or obstetrics and gynecology.

**Conclusions:**

Our results suggest that measures of physician training and experience are associated with the likelihood of Caesarean Section complications. In the context of a rural province deciding on the number of rural hospitals to keep open, this suggests a trade off between the benefits of increased volume versus the increased travel time for patients.

## Background

A large volume of literature has established that for some procedures, higher hospital and/or higher physician surgical volumes are associated with lower postoperative mortality or other adverse outcomes [1–3]. By comparison, the volume-outcome relationship is less well-established for Caesarean section surgeries (henceforth C-Sections). Of the research examining the relationship between physician characteristics and C-Sections, much of the focus is on the likelihood that a C-Section was performed (that is, instead of a vaginal delivery) [4–10].

A few international studies have reported a negative relationship between the likelihood of postoperative C-Section complications and years of surgeon experience [11, 12] and the surgeon’s C-Section volume [13]. On the other hand, neither the surgeon’s training level nor the surgeon’s case volume were found to be significant predictors of utero-cervical lacerations or blood loss in a study of anesthesia-related complications after C-Sections in New York State [14]. There is some evidence that the level of service at a hospital plays a role, with large variations in the probability of complications across US hospitals [15] and evidence that rural Canadian hospitals have poorer outcomes compared to larger ones [16]. There are very few studies of C-Section outcomes that adjust simultaneously for physician *and* hospital volume, and to our knowledge, none using Canadian data.

In this study, we aim to help fill this information gap by measuring associations between post-operative C-Section complications and physician and hospital characteristics at a population level. Echoing the trend in increasing C-Section rates worldwide, surgical deliveries have risen steadily in New Brunswick, Canada in recent years, currently representing 4.9% of all inpatient surgeries [17]. Our objective is to determine what factors are related to any observed volume-outcome relationships, that is, specific characteristics of the hospitals or physicians themselves, adjusting for measures of obstetric risk. We use a binary outcome variable coded as 1 if the mother suffers from any of four types of complications during her hospital stay. We use a population-based dataset of hospital discharge records from the province of New Brunswick. Due to Canada’s universal single-payer healthcare system that covers all physician and hospital services for residents, these data are considered population representative and complete.

The New Brunswick context is one of a relatively large rural population (48% rural compared to a national average of 19%, according to 2011 census data [18]), declining fertility (below-replacement total fertility rate of 1.60 in 2014 [19]), and low population density (10.5 residents per square kilometre [18]). There are currently 13 hospitals where women can give birth in the province. Few give birth outside the medicalized system [20]. For the province as a whole, approximately 27% of births in 2012 were via C-Section, and the primary C-Section rate is approximately 19% [21]. Ongoing debates on healthcare reform often raise the issue of closing smaller local hospital facilities in favour of larger regional hospitals (i.e. regionalization or centralization) due to the suggestion that quality of surgical outcomes may improve because of increased experience of surgeons and their teams. This research aims to strengthen the evidence base to inform health policy decisions.

## Methods

This study was approved by the University of New Brunswick (UNB) Research Ethics Board (REB file 2015–106). The analysis was conducted at the New Brunswick Institute for Research, Data and Training (NB-IRDT), a research institute on the UNB campus that provides approved researchers with secure access to de-identified health-related and other administrative databases. Our retrospective population-based analysis is based on Discharge Abstract Database (DAD) records, which have been linked to Provider Registry Data (for physician characteristics) and to Citizen Registry data (to determine patient characteristics including age, sex and six-digit residential postal code). Postal code information was further matched to Statistics Canada census data for information on socio-demographic characteristics of subjects’ home communities, including population size and average income levels. Data manipulation and preparation were performed using SAS software, while statistical and regression analyses were performed using MatLab.

### Cohort identification

We identify all hospital admissions (*n* = 20,914 admissions) for Caesarean section surgeries (Canadian Classification of Health Interventions code 5.MD.60.XX) in the province of New Brunswick, Canada, during the period from April 2004 to March 2014.

### Outcome variable

Among these patients, we identified those having any of the following four groups of complications related to their surgical delivery^1^:

- Postpartum hemorrhage – International Classification of Diseases (ICD, version 10) code O72;
  - Defined as: “Excess blood loss from uterine bleeding associated with obstetric labor or childbirth. It is defined as blood loss greater than 500 ml or of the amount that adversely affects the maternal physiology, such as blood pressure and hematocrit …. Hemorrhage defined as blood loss in excess of 500 ml after vaginal delivery or more than 1000 ml after a Caesarean section.” (icd10data.com)
- Postpartum sepsis – ICD code O85;
  - Includes postpartum sepsis, puerperal peritonitis, puerperal pyemia
- Postpartum infection – ICD code O86; and,
  - Includes: surgical wound complications and infections, other infections of genital tract, urinary tract infection, pyrexia of unknown origin, and other specified puerperal infections.
- Disseminated intravascular coagulation (DIC)– ICD-10 code D65.^2^

We coded the maternal complications outcome variable as 1 if any of the above complications followed surgery during the hospital stay, and 0 otherwise.

### Independent variable definitions

For each C-Section surgery, we identified the responsible physician (using the masked physician number) listed in the discharge abstract record as being the physician most responsible for the surgical procedure.^3^ We summed the number of C-Sections performed and defined physician caseload volume as the average annual number of surgeries performed by the physician over the previous 2 years (i.e., 730 days).^4^ After matching to physician registry records, we subtracted the year of the physician’s graduation from medical school from the year of the surgery to identify the number of years that the physician has been practising. We also identified physician specialization from the Physician Registry and identified those having specializations related to childbirth (i.e. maternal-fetal medicine or obstetrics and gynecology) versus all other specializations (who tend to be general surgeons).

We summed the number of C-Sections performed in each hospital and calculated a continuous variable indicating the average annual number of surgeries for each hospital. We created a categorical variable, separating the four higher-volume hospitals in New Brunswick’s larger cities (Fredericton, Moncton (2), and Saint John) from the nine lower volume hospitals in the rest of the province.

We also included patient characteristics and indications that might be associated with risk of complications. These include maternal age, whether the admission was deemed elective or urgent, and comorbidity indices (ranked from 0 to 4).^5^ Given the increasing mean maternal age and rising prevalence of chronic conditions and obesity in the general population, a product of which is an increasing probability that women with diabetes are becoming pregnant, or that gestational diabetes will develop, we further considered a previous diagnosis of diabetes as a pre-existing intraoperative or postoperative obstetric risk [22]. We created a variable indicating whether the patient has at any point prior to the admission (over our 10 years of data) had a diagnosis of diabetes (ICD-10 codes E10-E14, O24, P70.0, P70.1, R73.0).

Finally, we used the patient’s residential postal code matched to census data to assign the income quintile and population size of the community in which she resided at time of admission. Many studies have examined sociodemographic factors associated with C-section delivery, including income and living in an urban area [23]. Given the absence of personal income information in the provincial health administrative databases, we consider a proxy socioeconomic variable of neighbourhood income quintiles: a household size-adjusted measure of household income at the dissemination area level (the smallest standard geographic area) drawing on 2006 census data. The population size variable is based on 2011 census data. In New Brunswick, three categories are included: urban centres with populations between 100,000 and 499,000; small towns with populations between 10,000 and 99,999; and rural areas with populations lower than 10,000 people.

### Statistical analysis

We used logistic regression to identify the odds of maternal health complication occurring after C-Section surgery. First, we adjusted our statistical model for physician characteristics (i.e. case volume, experience, and specialization), patient characteristics and indications (i.e. age, previous diagnosis of diabetes, comorbidity level, and elective versus urgent admission category), and calendar year. Next, we adjusted additionally for the contextual characteristics (i.e. community size and income quintile). Third, we also adjusted for hospital volume. In order to test the robustness of our results on provider volume, we also ran models in which we included provider volume as a categorical variable.

## Results

We analyzed data for 20,914 admissions for C-Sections in New Brunswick, Canada, between the period of April 2004 and March 2014. Approximately 71% of admissions occurred in the four higher-volume hospitals, while the rest occurred in the remaining lower-volume hospitals (Table 1). Mean maternal age was 29 years and was roughly the same across higher- and lower-volume hospitals. The large majority of C-Sections admissions were deemed to be elective,^6^ while 10.6% were urgent. C-Sections performed in high-volume hospitals were more likely to be deemed urgent. Patients presenting at the higher-volume hospitals were more likely to have only mild or moderate comorbidities and were slightly less likely to have a diagnosis for diabetes than those presenting at lower-volume hospitals.

**Table 1:** Characteristics of patients admitted for Caesarian section surgery in New Brunswick (April 2004 – March 2014)

Characteristic	Lower-volume hospitals (*n* = 9)	Higher-volume hospitals (*n* = 4)	Total
Number of admissions	6096	14,818	20,914
Mean maternal age	29	30	29
Admission status (% of admissions)			
Elective	91.9	88.4	89.4
Urgent	8.1	11.6	10.6
Comorbidity (% of admissions)			
None (0)	53.3	52.5	52.7
Mild (1, 2)	7.9	9.7	9.2
Moderate to severe (3, 4)	1.2	2.3	2.0
Missing / not applicable	37.6	35.6	36.1
Diabetes in pregnancy (% of admissions)			
Diabetes mellitus or gestational diabetes	8.8	7.5	7.9
Community size classification (% of admissions)			
City (population 100,000 – 499,999)	0.2	50.2	35.6
Small town (population 10,000-99,999)	41.2	20.9	26.8
Rural area (population < 10,000)	58.6	28.9	37.5
Neighbourhood income quintile (% of admissions)			
Lowest	25.1	19.4	21.1
medium-low	21.7	18.8	19.6
Middle	19.9	19.3	19.5
Medium-high	17.4	19.9	19.1
Highest	15.7	22.6	20.6
Unknown	0.2	0.1	0.1
Physician years since graduation (% of admissions)			
0–9	7.0	21.4	17.2
10–19	30.9	38.2	36.1
20+	62.1	40.4	46.7
Physician specialization (% of admissions)			
Maternal-fetal or obstetrics & gynecology	82.2	99.8	94.6
All other specializations	17.8	0.2	5.4
Physician C-section annual volume (% of admissions)			
1–24	34.9	18.1	23.0
25–49	43.4	28.7	33.0
50–74	18.4	35.6	30.6
75–120	3.2	17.5	13.4
Maternal postoperative outcomes (% of admissions)			
Presence of complications	2.4	2.7	2.6

Patients presenting in the high-volume hospitals were considerably more likely to reside in cities than those presenting at low-volume hospitals. These patients were also more likely to come from neighbourhoods with higher income levels.

Over 62% of surgeries in the lower-volume hospitals were conducted by physicians who had more than 20 years since graduation, compared to only about 40% among those in the higher-volume hospitals. This finding generally corresponds to physicians in rural areas being older than their more urban peers. Nearly all C-Sections in higher-volume hospitals are performed by surgeons specialized in either maternal-fetal medicine or obstetrics and gynecology. In comparison, in lower-volume hospitals, nearly 18% of C-Sections are performed by surgeons with specializations other than maternal-fetal medicine or obstetrics and gynecology (these tend to be general surgeons). Not surprisingly, surgical volumes of providers were higher in high-volume hospitals than in low-volume hospitals. We observed complications in 2.6% of all surgeries, with a slightly higher (unadjusted) percentage in high-volume hospitals.

Our database contains details on 148 providing physicians, of whom 94 have provided at least one C-Section at a higher-volume hospital and 73 of whom performed at least one at a lower-volume hospital (Table 2).

**Table 2:** Characteristics of hospitals with relatively low and high volumes for C-section in New Brunswick (April 2004 – March 2014)

Characteristic	Lower volume hospitals	Higher volume hospitals	Total
Hospitals (n)	9	4	13
Providers (n)^a^	73	94	148
Annual hospital volume (mean)	95.4	332.4	263.3
Annual provider caseload (mean)	34.0	50.7	45.9

^a^Note that a given provider can perform surgeries in multiple hospitals, thus the sum of providers working in low and high volume hospitals is greater than the total number of providers

We present the results of our regressions in Table 3. Maternal age was inversely associated with likelihood of complication, although falls just short of statistical significance in two of three models. Our results suggest that a diagnosis of diabetes during pregnancy may be protective against the likelihood of complication, but these effects were not statistically significant in any of the three models. Not surprisingly, patients with one or more comorbidities were more likely to suffer from complications than those without comorbidities. Controlling for neighbourhood characteristics shows that patients living in wealthier neighbourhoods were less likely to suffer complications as a result of their surgery. Similarly, patients residing in more urban areas were also less likely to suffer complications. In model 3, we controlled for the mean annual hospital volume, which we found to be weakly positively associated with the likelihood of complications.

**Table 3:** Odds ratios (ORs) and 95% confidence intervals (CIs) for likelihood of maternal post-operative complication after Caesarean section (*n* = 20,914 patients)

	Model 1^a^			Model 2			Model 3		
	OR	95% CI		OR	95% CI		OR	95% CI	
Provider case volume	0.995	0.992	0.999	0.996	0.992	1.000	0.995	0.991	0.999
Provider years since graduation	0.965	0.956	0.974	0.962	0.953	0.972	0.965	0.956	0.975
Provider not specialized in maternal-fetal medicine or obstetrics & gynecology	1.424	1.006	1.842	1.443	1.013	1.872	1.588	1.149	2.026
Patient age (years)	0.984	0.969	1.000	0.987	0.971	1.003	0.987	0.971	1.003
Diabetes in pregnancy	0.912	0.593	1.230	0.884	0.565	1.203	0.880	0.561	1.200
Admission category elective (vs. urgent)	0.800	0.542	1.058	0.817	0.558	1.076	0.830	0.571	1.090
Patient comorbidity (level vs. none)									
1	3.420	3.157	3.683	3.415	3.152	3.679	3.397	3.133	3.661
2	1.647	1.168	2.127	1.666	1.185	2.146	1.664	1.183	2.145
3	2.517	1.960	3.074	2.621	2.063	3.179	2.616	2.058	3.174
4	4.866	4.085	5.647	5.148	4.363	5.932	5.025	4.239	5.810
Missing/not applicable	1.846	1.161	2.531	1.898	1.212	2.584	1.896	1.210	2.583
Neighbourhood income quintile (vs. lowest)									
Medium-low				1.022	0.764	1.280	1.025	0.767	1.283
Middle				0.917	0.649	1.186	0.918	0.649	1.186
Medium-high				0.884	0.612	1.156	0.883	0.610	1.155
Highest				0.887	0.617	1.156	0.880	0.610	1.150
Community size ( vs. city (population 100,000-499,000))									
Small town (population 10,000-99,999)				1.839	1.617	2.062	1.844	1.622	2.066
rural area (population < 10,000)				1.466	1.246	1.686	1.508	1.288	1.729
Hospital mean annual C-Section volume							1.001	1.000	1.002

^a^All regression models adjust for the listed variables and for a set of dummy variables for each year of the data period (2004–2014)

Provider volume was negatively associated with patient complication, regardless of the model; in all cases, patients of doctors with higher volumes had lower odds of complication. The odds ratio indicates that for a one unit increase in caseload, the likelihood of complication decreases by 0.5%, which corresponds to a decrease of 4.5% for a 10 unit increase in annual caseload. Physician experience, as measured by years since graduation from medical school, was also negatively correlated with complications; patients of physicians who have been practising for one additional year were 3.5% less likely to suffer from complications (16.3% less likely for a 5 year increase in years of experience). The provider specialization was also associated with the likelihood of complication; patients of physicians whose specialization is other than maternal-fetal medicine or obstetrics and gynecology were more likely to suffer complications. As mentioned previously, nearly all C-Sections occurring in higher-volume hospitals (i.e. the urban hospitals) are conducted by a physician specialized in maternal-fetal medicine or obstetrics and gynecology. However, roughly 18% of C-Sections in lower-volume (i.e. rural) hospitals are conducted by physicians with other specialities.

In Table 4, we allow for non-linear effects of physician volume by grouping physicians having average annual volumes from 1 to 24, 25–49, 50–74 and greater than 74. Based on the point estimates of the odds ratios, it seems that most of the decline in odds of complication occurs once physicians provide 50 or more C-Sections per year. However, at the 5% level, none of the categorical variables are significantly different from physicians performing from 1 to 24 surgeries per year.

**Table 4:** Odds ratios (ORs) and 95% confidence intervals (CIs) for likelihood of maternal post-operative complication after Caesarean section with provider case volume categories – nonlinear volume effects (*n* = 20,914 patients)

	Model 1^a^			Model 2^b^			Model 3^c^		
	OR	95% CI		OR	95% conf. Int.		OR	95% CI	
Provider annual volume (vs. 1–25)									
25–50	1.135	0.895	1.376	1.098	0.857	1.339	1.078	0.836	1.321
50–75	0.804	0.528	1.080	0.818	0.542	1.094	0.771	0.489	1.053
75+	0.802	0.458	1.146	0.851	0.506	1.196	0.762	0.404	1.120
Provider years since graduation	0.965	0.956	0.975	0.963	0.954	0.972	0.966	0.956	0.975
Provider not specialized in maternal-fetal medicine or obstetrics & gynecology	1.446	1.029	1.864	1.455	1.026	1.884	1.618	1.177	2.058

^a^Adjusted for patient age, diabetes status, elective/urgent admission status, and comorbidity and annual dummy variables

^b^Adjusted additionally for neighbourhood characteristics (income quintile and population size)

^c^Adjusted additionally for hospital volume

We also ran separate regressions for higher- and lower-volume hospitals individually. Results of these models were very similar to those already presented and to each other (not shown). Results were also robust to alternative models (not shown) where we categorized physicians’ years since graduation as 0–9 years, 10–19 years and 20+ years. These models showed monotonically decreasing odds ratios with increased years of experience. Finally, in order to attempt to exclude physicians visiting from other provinces (e.g. locums), for whom we likely undercount volume, we ran models excluding surgeries where the provider had fewer than 2 surgeries in the past 2 years. Findings were unchanged (results not shown).

## Discussion

In this whole-population study capturing information on roughly 21,000 C-section surgeries in New Brunswick, Canada, we found statistically significant associations between measures of physician training and experience and maternal post-operative complications. Specifically, we found that each of: physician C-section caseload volume, physician years of experience, and physician specialization in maternal-fetal medicine or obstetrics and gynecology, was associated with decreased odds of surgical complication. Of particular importance is the specialization of the physician. While the large majority of C-sections are performed by physicians specialized in maternal-fetal medicine or obstetrics and gynecology, our estimates suggest that surgeries performed by physicians with other specializations result in 40–50% more maternal complications. These associations were of a similar magnitude regardless of whether we adjusted for hospital volume or not.

Our associations between volume and adverse surgical outcomes were broadly consistent with the general literature on the volume-outcome relationship [1–3], as well as with the much smaller set of studies examining volume-outcome relationships for C-section surgeries specifically [11–13].

Inconsistent with the broader literature (see for example, [24]) is our finding of a positive association between hospital volume and complications, albeit one that is barely statistically significant. Few studies, however, are able to control separately for both hospital and physician volumes, and fewer also control for physician experience and area of specialization. These are notable strengths of this study.

One interpretation of our findings is that, at least for C-section surgery, it is primarily the physician’s experience that is important in determining patient outcomes, such that after the physician attributes are well controlled for, there is little volume-outcome relationship remaining at the hospital level. Another possible explanation is that there are not enough hospitals (*n* = 13) in our dataset for accurate inferences regarding hospital volume to be drawn.

Also consistent with the broad literature are our findings with respect to patient characteristics and indications. As is commonly found, patients with comorbid conditions, medically indicated surgical deliveries, and patients residing in low-income and more rural areas all face greater odds of C-section complications [25, 26]. The size of the odds ratios suggest that these patient-level effects are more important predictors of these outcomes than are physician or hospital volume. Thus, while our focus is on understanding the physician’s role in patient outcomes, it should not be forgotten that the pre-existing health condition of the patient is the most important determinant of their ultimate surgical outcome.

Key strengths of this study are that we have a large dataset linked to physician and patient information. We were able to adjust separately for measures of physician experience and hospital volume. Most previous studies of the volume-outcome relationship focus only on one of physician or hospital volume. Thus, we make a contribution by separately measuring the effect of physician volume, physician years of experience, physician specialty, and hospital volume. To our knowledge, this is the only Canadian study on the volume-outcome relationship for C-sections.

A limitation of this study was that we can only measure interventions performed in New Brunswick, by New Brunswick based providers, on New Brunswick residents. That is, we lacked information on providers who performed procedures in other provinces, and thus are not able to measure their volume completely if they performed surgeries in other provinces (although years since graduation and area of specialty would be measured accurately). However, our results were robust to excluding surgeries conducted by surgeons with very low measured volumes. Other limitations included using area-level socioeconomic measures because individual-level maternal socioeconomic information were not available in the data. An additional limitation is that our estimate of years of experience does not capture career interruptions (e.g., parental leave), so our measure of experience should be considered an upper bound. Finally, hospital discharge abstract lack some contextual information which might confound the analysis. For example, the data do not capture information on whether the C-Section is elective, although we understand that very few are. Further, the data do not contain information on the duration of the labour prior to the C-section nor whether the C-section was conducted prior to the onset of labour.

A final limitation is that our database contains only limited information on the complexity of the specific patient’s case (i.e. comorbidity index, age). It is therefore possible that more complicated cases (on dimensions unobserved in the database) are allocated to more experienced physicians or higher-volume hospitals, and this could be confounding our analysis. We believe that this is likely an unusual occurrence due to the distance between hospitals. To the extent that it occurs, however, we are likely underestimating the impact of a surgeon’s experience on reducing complications – that is, had they not had the most difficult cases, the more experienced / high caseload physicians might have had even lower rates of complication.

## Conclusion

This study showed that the likelihood of maternal post-surgical complications after Caesarean Section was negatively associated with a series of measures of the surgeon’s characteristics (e.g. volume, years of experience, and surgical specialty). This finding has particular relevance in rural areas, such as New Brunswick, where policy-makers must decide on the number of hospitals to open in rural areas. Increasing the number of hospitals therefore likely implies a trade-off between desirable shorter travel distances for patients but undesirable lower volumes for providers.

Future studies on this topic may wish to explore other indicators of provider experience, including longer-term estimates of volume, as well as the role of individual-level socioeconomic characteristics of patients in modifying the likelihood of post-C-section complications.

## Abbreviations

CIHR: Canadian Institutes of Health Research
C-Sections: Caesarean Section surgeries
DAD: Discharge Abstract Database
DIC: Disseminated Intravascular Coagulation
ICD: International Classification of Diseases
MSSU: Maritime SPOR (Strategy for Patient-Oriented Research) Support Unit
NBHRF: New Brunswick Health Research Foundation
NB-IRDT: New Brunswick Institute for Research, Data, and Training
NSHRF: Nova Scotia Health Research Foundation
REB: Research Ethics Board
UNB: University of New Brunswick

## Footnotes

1 Complications were based on ICD 10 classifications. Complete listings of ICD 10 codes are available from the World Health Organization at: http://apps.who.int/classifications/icd10/browse/2016/en.

2 DIC is an extremely rare complication in our dataset with fewer than 5 occurrences. Results are robust to its inclusion or exclusion.

3 There may be multiple procedures recorded in the discharge abstracts for which there may be multiple doctors (i.e. there may be both a delivering and discharging physician). We attribute experience in the C-Section only the physician associated with the C-Section procedure code (i.e. the delivering physician).

4 The intent of this variable was to capture current caseload volume as compared to total experience (which is measured separately as years since graduation). A period of 2 years was selected to ensure that temporary declines in activity (e.g. vacations or maternity leaves) did not unduly lower our measure of current caseload.

5 The comorbidity level reflects the cumulative resource impact of comorbidities on the patient’s stay, as assigned by the Canadian Institute for Health Information’s (CIHI) Case Mix Grouping methodology. These are comparable to Diagnosis Related Groups in the US. More information at www.cihi.ca.

6 Note that the admission status (elective vs. urgent) is determined at the time of arrival at the hospital. It is distinct from whether the C-section itself was elective or medically indicated, and we do not have data on the latter (and the number of elective C-sections is believed to be very small).
